# What does the multiple mini interview have to offer over the panel interview?

**DOI:** 10.3402/meo.v21.29874

**Published:** 2016-02-11

**Authors:** Allan Pau, Yu Sui Chen, Verna Kar Mun Lee, Chew Fei Sow, Ranjit De Alwis

**Affiliations:** 1 Division of Community and Child Oral Health, School of Dentistry, International Medical University, Kuala Lumpur, Malaysia; 2 Division of Human Biology, School of Medicine, International Medical University, Kuala Lumpur, Malaysia; 3 Department of Family Medicine, School of Medicine, International Medical University, Kuala Lumpur, Malaysia; 4 Clinical Skills and Simulation Centre, School of Medicine, International Medical University, Kuala Lumpur, Malaysia; 5 Department of Community Medicine, School of Medicine, International Medical University, Kuala Lumpur, Malaysia

**Keywords:** multiple mini interview, panel interview, students selection, students admission

## Abstract

**Introduction:**

This paper compares the panel interview (PI) performance with the multiple mini interview (MMI) performance and indication of behavioural concerns of a sample of medical school applicants. The acceptability of the MMI was also assessed.

**Materials and methods:**

All applicants shortlisted for a PI were invited to an MMI. Applicants attended a 30-min PI with two faculty interviewers followed by an MMI consisting of ten 8-min stations. Applicants were assessed on their performance at each MMI station by one faculty. The interviewer also indicated if they perceived the applicant to be a concern. Finally, applicants completed an acceptability questionnaire.

**Results:**

From the analysis of 133 (75.1%) completed MMI scoresheets, the MMI scores correlated statistically significantly with the PI scores (*r*=0.438, *p*=0.001). Both were not statistically associated with sex, age, race, or pre-university academic ability to any significance. Applicants assessed as a concern at two or more stations performed statistically significantly less well at the MMI when compared with those who were assessed as a concern at one station or none at all. However, there was no association with PI performance. Acceptability scores were generally high, and comparison of mean scores for each of the acceptability questionnaire items did not show statistically significant differences between sex and race categories.

**Conclusions:**

Although PI and MMI performances are correlated, the MMI may have the added advantage of more objectively generating multiple impressions of the applicant's interpersonal skill, thoughtfulness, and general demeanour. Results of the present study indicated that the MMI is acceptable in a multicultural context.

Admission to medical schools is highly competitive. Pre-admission academic ability is a prerequisite universally as it is the best predictor of academic performance during training, although its relationship with clinical performance is less clear (1, 2). As the number of academically able applicants exceeds the number of training places available, additional measures such as the aptitude test, personal statement, and interview are used. The admission interview is used to determine whether candidates possess the requisite personal traits suitable for medical training and effective communication skills, and it also serves as an opportunity to examine whether prospective students are truly interested in medicine and the particular school applied for.

The panel or board interview is commonly used in addition to pre-admission academic qualifications to aid decisions (3). Students selected by the interview have been reported to perform better, for example, in communications Objective Structured Clinical Examination (OSCE), compared with those selected by academic merit alone (4). However, the reliability and validity of the panel interview (PI) have been increasingly questioned (5, 6). Structuring the interview has been reported to enhance its acceptability and reliability (7). The multiple mini interview (MMI) has been designed to be highly structured, resembling the OSCE (8).

In the MMI, candidates rotate around a series of short interviews that assesses non-cognitive attributes currently assessed in the PI. The stations are usually designed in such a way that they do not require any specific learned knowledge but rather evaluate the applicant's ability to logically work through a problem and express their ideas clearly. The stations may be designed to involve discussing one's viewpoint on a particular social ethical dilemma with one or more faculty or lay interviewers, role-playing with an actor or simulated patient, or completing a specific task. Candidates may be assessed for critical thinking, communication skills, and ability to coherently discuss particular social dilemmas. It is suggested that the MMI allows a wide sampling of applicants’ competencies so that a more accurate picture of their overall ability may be assessed. Furthermore, in some MMIs, interviewers are asked to raise a ‘red flag’ or indicate a ‘concern’ if they felt the applicant was behaving inappropriately at an MMI station, that is, overly aggressive, timid, rude, immature, etc.

Typically the MMI consists of 10 stations, each lasting 8 min and assessed by one interviewer, and has been reported to be reliable, acceptable and feasible (9). The multiple assessments of applicants by different interviewers across different domains increase the reliability of the MMI. Compared to the autobiographical sketch, pre-admission academic qualifications, aptitude test and PI, performance at the MMI has been reported to be a statistically significant predictor of success in early years at medical school training (10, 11). Applicants who performed well at the MMI have also been reported to do better at national licensing examinations (12). Medical schools that have documented using the MMI have reported that it is acceptable (13–15) and feasible (16). However, most reports of MMI have been from the Western experience. Its acceptability in other contexts has rarely been reported.

Although the rise in the use of MMIs shows that the traditional PI is now becoming outdated as a method to be used in isolation, continuous research is necessary to further build the evidence base to show that the MMI is the best way to select future doctors. In this paper, we compare the PI performance of a sample of medical school applicants to their MMI performance, and evaluate the acceptability of the MMI in a multiracial and non-Western context.

## Methods

### Sample selection

Ethics approval was obtained from the Institutional Research and Ethics Committee. Applicants in an MBBS programme in one medical school shortlisted for the PI were invited to participate in an MMI pilot. A letter of invitation and an information leaflet on the MMI process were given to potential participants via email. The applicants were assured that the outcome of the MMI would not affect their selection for entry into the medical programme. Selection was based on academic performance and the PI. Confidentiality of MMI and PI performances was assured. Written consent was obtained from the applicants. This also included consent for the use of examination results for the purpose of data analysis. Participation was on a voluntary basis. The interviews were conducted during September/October 2013. Interviewers were recruited from academic faculty. They were given a one-day training session and an MMI assessment manual. On the days of the interviews, a reminder briefing was given.

### Data collection

Those applicants who consented to participate attended on the days that both the PI and MMI were administered. Each applicant was first interviewed by a panel of two faculty interviewers for 30 min. Five panels interviewed one applicant each concurrently for 30 min, resulting in 10 applicants interviewed in 60 min. After the PI, the 10 applicants were briefed for 30 min on the MMI before they participated in the MMI. The MMI stations’ interviewers were different from those who had taken part in the PI. This was to avoid the influence of preconceived impressions of the applicants generated in the PI.

The MMI consisted of 10 stations each of 8 min, including 2 min of preparation time. Each station was assessed by one faculty interviewer. One station required the applicant to complete a task with a helper, two required the applicant to role-play with an actor, and the remaining seven stations consisted of one-to-one interviews. Each station tested a specific attribute and the attributes tested were: ability to be a team player, conscientiousness, altruism, ability to cope with stress, ability to summarise, decision-making skills, tolerance and adaptability, empathy, career motivation, and honesty and integrity.

Data were collected on age, sex, ethnicity, and academic qualifications at entrance. The various pre-admission qualifications were banded to categorise academic ability, that is, Band 1 – higher academic ability, Band 2 – moderate academic ability, and Band 3 – lower academic ability. This was necessary so that applicants may be compared using a single classification system as the pre-admission qualifications of applicants were rather diverse, such as ‘A’ levels, the national sixth form qualification, the International Baccalaureate, and the Australian and Canadian matriculation. During the PI, each interviewer assessed the ‘personal characteristics’, ‘desirable attitudes and values of the future healthcare professional’, ‘appropriate skills for the future healthcare professional’, ‘ability to make decisions and tolerate uncertainty’, and ‘communication skills’. A total maximum score of 20 was possible. The average of the two interviewers was taken as the final score. In the MMI, the score at each station was standardised to 10, giving a possible total maximum score of 100. At each station the interviewer was asked to indicate a concern if the subject was overly aggressive, timid, arrogant, rude, immature, etc. The subjects were categorised into the number of stations at which they were indicated as a concern. At the end of the MMI, the subjects were asked to complete an anonymous questionnaire on the acceptability of the MMI. Examples of questions asked were, ‘I received adequate information prior to my interview about the MMI’, ‘The pre-MMI briefing on the day prepared me for the MMI’, and ‘The instructions at the MMI stations were clear and easy to understand’. Each question was scored on a 5-point Likert scale from ‘Strongly Disagree’ (1) to ‘Strongly Agree’ (5).

### Data analysis

The mean PI and MMI scores with 95% confidence intervals (CIs) were calculated and compared by age, sex, race, and academic ability using *t* test and ANOVA. The number of stations at which subjects were indicated as a concern was cross-tabulated against age, sex, race, and academic ability for association using the chi-square test. Pearson's correlation analysis was carried out to test for association between PI and MMI. The *t* test was carried out for association between number of stations indicated as concern and PI and MMI scores. Frequency distributions for acceptability questions were calculated. The mean score for each item was tested for association with sex and race using the *t* test.

## Results

Of 177 participating applicants, 131 (75.1%) MMI scoresheets were fully completed. Seventy-eight (59.5%) subjects were females compared with 53 (40.5%) males, and 109 (83.2%) subjects were aged 19 or younger and 22 (16.8%) were aged 20 or older (Table 1). Ninety-two (70.2%) subjects were Chinese, whereas 12 (9.2%) were Malay, 18 (13.7%) were Indian, and 9 (6.9%) were others. Academically able (Band 1) subjects numbered 70 (53.4%) compared with 23 (17.6%) moderately able (Band 2) and 38 (29.0%) less able (Band 3) subjects. The mean PI score was 15.7 (95% CI 15.2–16.1) and mean MMI score was 72.5 (95% CI 70.8–74.1), both of which were not statistically significantly associated with sex, age, race or academic ability. One-hundred-and-twenty (91.6%) subjects were assessed as a concern at one station or not at all, whereas eleven (8.4%) were assessed as a concern at two or more stations (Table 1). Assessment of a concern was not statistically significantly associated with sex, age, race, and academic ability.

**Table 1:** Comparison of mean PI and MMI scores with 95% confidence intervals by sex, age, race, and pre-university academic ability (*n*=131)

	*N* (%)	Mean PI score (95% CI)	*p* value	Mean MMI score (95% CI)	*p* value	Assessed as concern at one station or none *N* (%)	Assessed as concern at two stations or more *N* (%)	p value
Male	53 (40.5)	15.6 (14.9–16.3)	0.798	72.6 (69.7–75.5)	0.895	47 (88.7)	6 (11.3)	0.320
Female	78 (59.5)	15.7 (15.1–16.3)		72.4 (70.4–74.4)		73 (93.6)	5 (6.4)	
19 and younger	109 (83.2)	15.7 (15.3–16.2)	0.453	72.8 (71.1–74.6)	0.396	99 (90.8)	10 (9.2)	0.475
20 and older	22 (16.8)	15.3 (14.0–16.6)		70.9 (65.9–75.9)		21 (95.5)	1 (4.5)	
Chinese	92 (70.2)	15.4 (14.9–16.0)	0.367	71.8 (69.8–73.8)	0.102	81 (88.0)	11 (12.0)	0.165
Malay	12 (9.2)	15.5 (13.3–17.7)		69.2 (62.7–75.8)		12 (100.0)	0	
Indian	18 (13.7)	16.3 (15.3–17.4)		76.4 (72.6–80.2)		18 (100.0)	0	
Others	9 (6.9)	16.7 (14.4–18.9)		76.1 (70.6–81.6)		9 (100.0)	0	
Band 1 – higher academic ability	70 (53.4)	15.7 (15.0–16.3)	0.926	72.4 (70.2–74.6)	0.578	65 (92.9)	5 (7.1)	0.670
Band 2 – moderate academic ability	23 (17.6)	15.8 (14.8–16.9)		74.3 (70.5–78.0)		20 (87.0)	3 (13.0)	
Band 3 – lower academic ability	38 (29.0)	15.6 (14.7–16.4)		71.6 (68.1–75.2)		35 (92.1)	3 (7.9)	
Sample	131 (100.0)	15.7 (15.2–16.1)		72.5 (70.8–74.1)		120 (91.6)	11 (8.4)	

**Fig. 1:**
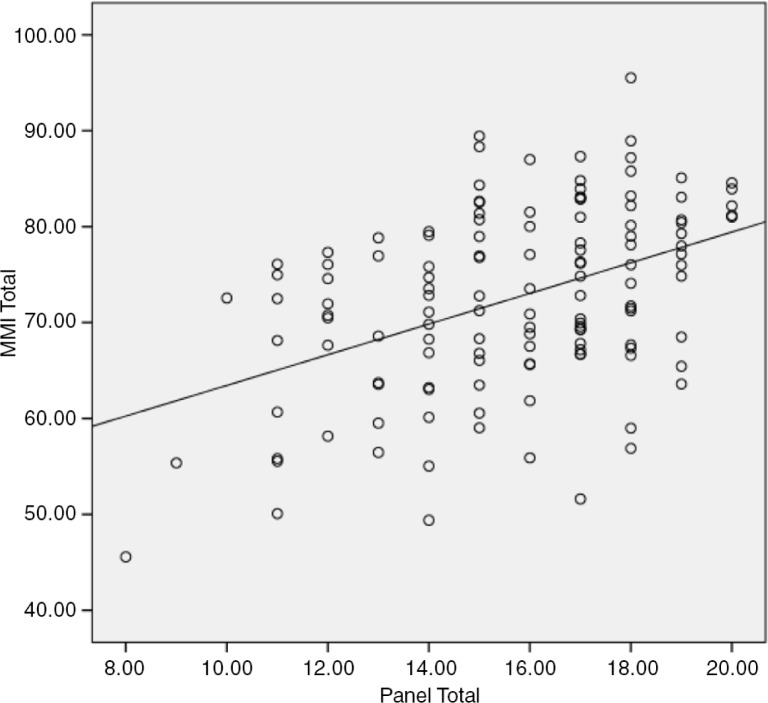
Correlation between PI and MMI scores: Pearson's correlation coefficient = 0.438 (*p*=0.001).

The correlation coefficient between total PI and MMI scores was 0.438 (*p*=0.001), although it should be noted that for any PI score, the range of MMI scores was quite wide (Fig. 1). For example, for the PI score of 15, the MMI scores ranged from 60 to 90.

The mean MMI score for those assessed as a concern at one station or none was statistically significantly higher at 73.5 (95% CI 71.9–75.1) compared with those assessed as a concern at two stations or more at 61.7 (95% CI 55.1–68.4, *p*=0.001) (Table 2). However, mean PI scores for both groups were not statistically significantly different.

**Table 2:** Comparison of mean PI and MMI scores (95% CI) by assessment of concern (*n*=131)

	Mean PI score (95% CI)	*p* value	Mean MMI score (95% CI)	*p* value
Assessed as concern at one station or none	15.7 (15.3–16.2)	0.268	73.5 (71.9–75.1)	0.001
Assessed as concern at two stations or more	14.8 (13.3–16.4)		61.7 (55.1–68.4)	

Most subjects strongly agreed (36.9%) or agreed (35.2%) that they received adequate information about the MMI prior to their interview (Table 3). However, relatively fewer strongly agreed (9.1%) or agreed (24.0%) that they used information from various sources to prepare for the MMI. Most strongly agreed (27.4%) or agreed (40.0%) that the pre-MMI briefing was helpful. The majority strongly agreed (30.7%) or agreed (51.7%) that the instructions at the MMI stations were clear. Similarly, subjects also strongly agreed (27.4%) or agreed (49.1%) that it was clear to them what the MMI was assessing. Over two-thirds strongly agreed (32.4%) or agreed (35.2%) that they enjoyed the MMI. Just over one-third reported that they strongly agreed (13.6%) or agreed (22.7%) that they felt stressed before the MMI. Almost all strongly agreed (59.1%) or agreed (30.1%) that the interviewers were polite and respectful during the MMI.

**Table 3:** Number of subjects (%) responding to the acceptability questions on the MMI (*n*=173)

	Strongly disagree	Disagree	Neither agree nor disagree	Agree	Strongly agree
I received adequate information prior to my interview about MMI	11 (6.3)	17 (9.7)	21 (11.9)	62 (35.2)	65 (36.9)
I used information from various sources to prepare myself for the MMI	33 (18.9)	30 (17.1)	54 (30.9)	42 (24.0)	16 (9.1)
The pre-MMI briefing on the day prepared me for the MMI	3 (1.7)	11 (6.3)	43 (24.6)	70 (40.0)	48 (27.4)
The instructions at the MMI stations were clear and easy to understand	1 (0.6)	4 (2.3)	26 (14.8)	91 (51.7)	54 (30.7)
It was clear to me what the MMI was assessing	1 (0.6)	9 (5.1)	31 (17.7)	86 (49.1)	48 (27.4)
I enjoyed the MMI	2 (1.1)	14 (8.0)	41 (23.3)	62 (35.2)	57 (32.4)
I felt stressed before the MMI	30 (17.0)	41 (23.3)	41 (23.3)	40 (22.7)	24 (13.6)
Interviewers or assessors were polite and respectful to me during the MMI		4 (2.3)	15 (8.5)	53 (30.1)	104 (59.1)

The mean scores for each of the acceptability items were compared between sex and race categories, but no statistically significant difference in scores were detected (Table 4).

**Table 4:** Comparison of mean scores (95% CI) for questions on acceptability of the MMI by sex and race

	Sex		Race			
		
	Male (*n*=65)	Female (*n*=108)	Malay (*n*=11)	Chinese (*n*=129)	Indian (*n*=26)	Others (*n*=7)
I received adequate information prior to my interview about the MMI	3.62 (3.31–3.92)	4.02 (3.80–4.24)	3.67 (2.84–4.49)	3.83 (3.62–4.04)	4.15 (3.73–4.58)	4.00 (2.69–5.31)
I used information from various sources to prepare myself for the MMI	2.88 (2.55–3.21)	2.87 (2.64–3.10)	2.83 (2.13–3.54)	2.90 (2.68–3.12)	2.88 (2.41–3.36)	2.29 (1.59–2.98)
The pre-MMI briefing on the day prepared me for the MMI	3.78 (3.55–4.01)	3.90 (3.71–4.08)	3.83 (3.24–4.43)	3.81 (3.63–3.98)	4.12 (3.83–4.40)	3.71 (2.69–4.74)
The instructions at the MMI stations were clear and easy to understand	4.17 (4.01–4.33)	4.05 (3.89–4.21)	4.33 (3.92–4.75)	4.05 (3.91–4.18)	4.19 (3.94–4.45)	4.14 (3.15–5.13)
It was clear to me what the MMI was assessing	4.03 (3.85–4.21)	3.94 (3.77–4.12)	4.00 (3.39–4.61)	3.94 (3.79–4.09)	4.19 (3.89–4.49)	3.71 (2.83–4.59)
I enjoyed the MMI	3.98 (3.77–4.20)	3.83 (3.63–4.03)	4.00 (3.34–4.66)	3.91 (3.74–4.08)	3.96 (3.56–4.37)	3.29 (2.26–4.31)
I felt stressed before the MMI	3.11 (2.79–3.42)	2.84 (2.59–3.09)	2.75 (2.03–3.47)	2.99 (2.76–3.22)	2.81 (2.31–3.31)	2.86 (1.22–4.50)
Interviewers or assessors were polite and respectful to me during the MMI	4.48 (4.29–4.66)	4.44 (4.30–4.59)	4.75 (4.46–5.04)	4.39 (4.25–4.53)	4.65 (4.43–4.88)	4.57 (3.84–5.30)

## Discussion

This study was carried out to compare the performance of a sample of medical school applicants at a PI and an MMI, evaluate the acceptability of the MMI in a multiracial context, and determine the logistic feasibility of the MMI compared to the PI. The key findings were that PI and MMI performances were generally not associated with sex, age, race, and entrance academic ability, PI and MMI scores are statistically significantly correlated, and assessment of a concern was not associated with PI scores. Most subjects reported that the MMI was acceptable, and there were no differences in acceptability scores.

The PI has often been criticised for being biased (3), but in this study no statistically significant differences were noted between applicants who were male or female, from different age or racial groups, or pre-admission academic ability. Similarly, consistent with previous reports, the results of the present study indicated that the MMI was not biased (8, 13, 16–18). This is not a universal observation as female applicants have been reported to perform better in an MMI (19) administered to a sample of dental students. However, it should be noted that this does not necessarily indicate bias in the MMI. The finding that PI and MMI scores were not associated with pre-admission academic ability is consistent with reports that the admission interview assesses non-cognitive attributes (8, 20). Less frequently some studies have reported association between academic ability and MMI performance in emergency medicine residents (21) and graduate-entry dental students (22).

After academic ability, the interview is the most commonly used method to assess the suitability of candidates for medical training (1). The PI is the usual practice, but the MMI is increasingly adopted because of its better reliability through its highly structured format and multiple assessments by different interviewers. However, the PI may be designed to be highly structured, such that differences in performances between the PI and MMI may be insignificant. The results of the present study indicated that the subjects who performed well in the PI were statistically significantly more likely to perform well in the MMI. This suggests that the MMI assessment of applicants was no different to the PI assessment and therefore does not add any more value to the PI. However, it should be noted that of all the applicants who performed well in the PI, for example, at a score of 15 and above, their MMI scores were dispersed considerably. Thus, although there was a statistically significant association between the PI and MMI, a significant minority of poor MMI performers scored high in the PI. If current evidence for the validity of the MMI is to be accepted (10, 12, 23), then a significant minority of unsuitable candidates would have been selected for medical training.

Perhaps the more significant contribution of the MMI is the generation of multiple impressions of the candidate's interpersonal skill, thoughtfulness, and general demeanour (12) to identify those who are unsuitable for medical training. And this may be generated in a relatively short period of time (24). Candidates who were raised as a concern independently at two stations or more performed similarly in the PI to those who were raised as a concern at one station or none at all. Although assessment of concern was subjectively determined at each station, the multiple independent assessments of applicants by different interviewers across different stations increase the reliability and objectivity of the assessment. This suggests that the MMI may have the added advantage of more objectively identifying applicants who may not be suitable to train as doctors because of particular concerns. An interesting finding was that all 11 applicants who were identified as a concern at two or more stations were of Chinese racial background. Comments made by the interviewers on their scoresheets suggested that they were either extremely nervous, overly arrogant, or inarticulate. It was noted that just over half of these applicants were raised as a concern at the *Teamwork* station, in which applicants were interviewed on their perceptions and experiences of teamwork. Although the data do not allow definitive conclusions, it may be postulated that the poor performance of the Chinese applicants at the *Teamwork* station in this context reflected their socially more competitive instincts compared to the other racial groups (25). This is explained by their perception of marginalisation as a minority group and their need to be competitive as individuals. Anecdotally, it has been observed that some Chinese students are less willing to share their learning in the classrooms. However, longitudinal follow-up of subjects assessed as a concern is necessary to determine if this assessment is predictive of future academic and clinical performance. The applicants admitted to the programme will be followed up, and their performance at the MMI will be assessed for association with future academic and clinical performance.

Consistent with previous reports, subjects in the present study generally rated the acceptability of the MMI positively. Performance at the MMI did not appear to favour subjects from particular sex, age, and racial groups, or academic ability. More importantly, responses to the acceptability questionnaire indicated that the MMI was acceptable irrespective of sex or race. Most published research on the MMI has emanated from English-speaking countries where the population might be considered to be more homogeneous on grounds of race, religion, and language than some parts of the world. Results of the present study indicated that the MMI is acceptable in a multicultural context.

The findings of this study should be considered in the context of its limitations. Firstly, identification of a concern can vary widely, especially in the context of multicultural and multinational interviewers at this university. However, as each subject was assessed independently at more than one station, better reliability may be expected. Identification as a concern does not necessarily mean that the subject will perform poorly during medical training or clinical practice. Longitudinal evaluation of this group of students is necessary to validate the usefulness of identifying candidates as being a concern.

## Ethical approval

This study was approved by the International Medical University Joint-Committee on Research and Ethics.

## Contributors

AP acted as a primary investigator and contributed to the conception and design of the study and the acquisition, analysis, and interpretation of data, and drafted the paper. VKML contributed to designing and vetting of the MMI stations and acquisition, analysis, and interpretation of data, and reviewed the manuscript. YSC contributed to the conception and design of the study and data acquisition, and reviewed the manuscript. CFS, RDA, and AAF contributed to designing and vetting of the MMI stations and acquisition of data, and reviewed the manuscript.
